# Birth of Cone Bipolar Cells, but Not Rod Bipolar Cells, Is Associated with Existing RGCs

**DOI:** 10.1371/journal.pone.0083686

**Published:** 2014-01-02

**Authors:** Ling Bai, Takae Kiyama, Hongyan Li, Steven W. Wang

**Affiliations:** 1 Department of Ophthalmology and Visual Science, University of Texas Health Science Center at Houston Medical School, Houston, Texas, United States of America; 2 Department of Ophthalmology, the Second Affiliated Hospital, School of Medicine, Xi'an Jiaotong University, China; Dalhousie University, Canada

## Abstract

Retinal ganglion cells (RGCs) play important roles in retinogenesis. They are required for normal retinal histogenesis and retinal cell number balance. Developmental RGC loss is typically characterized by initial retinal neuronal number imbalance and subsequent loss of retinal neurons. However, it is not clear whether loss of a specific non-RGC cell type in the RGC-depleted retina is due to reduced cell production or subsequent degeneration. Taking advantage of three knockout mice with varying degrees of RGC depletion, we re-examined bipolar cell production in these retinas from various aspects. Results show that generation of the cone bipolar cells is correlated with the existing number of RGCs. However, generation of the rod bipolar cells is unaffected by RGC shortage. Results report the first observation that RGCs selectively influence the genesis of subsequent retinal cell types.

## Introduction

The mammalian retina contains six major neuronal and one glial cell types. Among them, retinal ganglion cells (RGC), horizontal cells, amacrine cells and cone photoreceptors are born in the early phase of retinal neurogenesis and are regarded as embryonic or early cell types. Rod photoreceptors, bipolar cells and Müller glia are born in the late phase of retinal neurogenesis and are regarded as post-natal or late cell types. RGC, for its leading birth sequence, is believed to play an important developmental role in the formation of the remaining retina[1]. Dissociated cells from retinal primordia develop mostly into RGCs suggesting the existence of inhibitory factors to prevent further RGC formation in an developing retina[2]. Factors released by RGC that can influence further retina development have been identified thereafter. For example, GDF11 is released by newborn RGCs to preclude further RGC production from the progenitor pool and thus providing opportunities for the production of other cell types [3]. Most importantly, RGCs play a histogenic role by releasing Sonic Hedgehog (SHH) to stimulate progenitor proliferation and thus assure adequate retinal cell number [4]–[6]. RGC's histogenic role is further supported by a transcriptome screening effort in *Atoh7^−/−^* retinas that have 95% developmental RGC loss, in which expression of Gli1, a downstream effector to SHH signaling, is significantly reduced at stages of early retinal neurogenesis [7]. However, it is not known whether all subsequent retinal cell types rely equally on RGCs. Our earlier studies on the *Atoh7^−/−^* retina, which had only 5% of normal RGC number, showed unchanged rod bipolar cell (R-BPC) numbers implying production of R-BPCs is not affected in an RGC-depleted retina [8], [9]. In contrast, other studies reported that R-BPCs are significantly reduced in the *Atoh7^−/−^* retina suggesting either Atoh7 or RGCs are required for normal R-BPC production [10], [11]. To address this discrepancy, we revisited the *Atoh7^−/−^* retina by probing into the possibility of degenerative R-BPCs. Our results show that, in the *Atoh7^−/−^* retina, R-BPCs are generated in a normal number but degeneration become apparent after 1 month of age. Moreover, in the same retina, significantly fewer cone bipolar cells (C-BPCs) are generated, but their number remains stable throughout the adulthood. To investigate whether reduced C-BPC production is a cell autonomous or non-cell autonomous event, we further included two other retinas that had varying degree of RGC loss. Our results indicate that production of C-BPC is related to the existing number of RGCs, but production of R-BPC is independent from RGCs.

## Materials and Methods

### Ethics statement

Animal use in these experiments was approved by the Animal Welfare Committee of the University of Texas Health Science Center at Houston in accordance with the guidelines established by the National Institutes of Health. In the process of euthanasia, animals at or younger than postnatal day 7 (P7) were anesthetized using hypothermia prior to decapitation to reduce pain and eliminate suffering. Animals at or older than P10 were euthanized through cervical dislocation by highly trained personnel to ensure no suffering in the process.

### Animals and genotyping

Single knockouts of *Pou4f2* (*Brn3b^AP/AP^*) and *Atoh7* (*Math5^LacZ/LacZ^*) mice were established as described previously [8], [12]. They are designated as *Pou4f2^−/−^* and *Atoh7^−/−^* in this study as we did not utilize their knock-in reporter genes. Double knockout of *Atoh7* and *Pou4f2* (*Math5^LacZ/LacZ^;Brn3b^AP/AP^*) mice were generated previously in our laboratory [9]. This strain is designated as *Atoh7^−/−^;Pou4f2^−/−^* for the same reason. For simplicity, it is mentioned in the text as double knockout (DKO).

The *Atoh7^GFP/GFP^* and *Vsx1^LacZ/LacZ^* mice were crossed to generate an *Atoh7^−/−^*;*Vsx1^LacZ/LacZ^* line. The established line was then crossed with *Atoh7^+/−^* mice to create *Atoh7^−/−^;Vsx1^LacZ/+^* and *Atoh7^+/−^;Vsx1^LacZ/+^* pups for examining C-BPC subpopulations using *Vsx1^LacZ^* expression. Genotyping for *Vsx1^LacZ/LacZ^* was conducted as described previously [13].

### Determination of the retinal ganglion cell population

Numbers of RGCs in the *Pou4f2^−/−^* and *Atoh7^−/−^* retinas, were determined previously using various methods including 1,1′-dioactadecyl-3,3,3′,3′-tetramethylindocarbodyanine perchlorate (Dil) labeling by gene gun and retrograde labeling [14]. The counts showed 80% and 95% RGC loss in the *Pou4f2^−/−^* and *Atoh7^−/−^* retinas respectively. These numbers are slightly higher than the rough estimations reported previously [8], [15], which were obtained by counting all nuclei in the RGC layer as well as estimating RGC axon numbers. In an attempt to estimate the RGC number in the *Atoh7^−/−^;Pou4f2^−/−^* DKO retina, DiI gunning yielded no RGCs and there was no optic nerve, making retrograde dye tracing impossible. Therefore, RGC number was estimated by comparing neurofilament light chain (NF-L) positive cells in the RGC layer in flat-mounted retinas with that of *Atoh7^−/−^* retinas [9]. Estimates showed that each *Atoh7^−/−^;Pou4f2^−/−^* retina had less than one fifth of the number of NF-L-positive axons that were observed in the *Atoh7^−/−^* single mutant retina, which is corresponding to approximately less than 1% of normal RGC population. In this study, we designate the *Atoh7^−/−^;Pou4f2^−/−^* (DKO) retina as having 1% of normal RGC number for simplicity.

### Rationale on sampling stages for assessing proliferating cells

The main sampling stage for estimating the number of proliferating cells in this study was postnatal day 4 (P4). This stage was selected for the best avoidance of overlapping production time of bipolar cells from other cell types. All the early cell types, RGCs, horizontal cells, cones, and amacrine cells, have ceased production at P4; while BPC production remains at its height and production of rod photoreceptors has dropped significantly [16]. Three-day intervals following P4 were added (P7 and P10). And, to allow direct comparisons with our previous study [9], we included the earlier timepoint, P0.

### Sample collection, immunohistochemistry, & microscopy

Eyeballs were collected and fixed in 4% paraformaldehyde (PFA, EMS 15710) in phosphate buffered saline (PBS) for 2 hr, washed 3 times with PBS-T, infiltrated in 15% sucrose overnight, and embedded in O.C.T. (Tissue-Tek 4583). Radial cryosections were collected at 30 µm thickness and only three most center sections in each retina were used. Center of the retina was judged by their largest circumference and presence of optic disc. Occasional obliquely sectioned retinas, as judged by shorter soma and abnormally thick nuclear layers, were discarded.

Cryosections were blocked for 1 hr with 2% BSA 5% normal goat serum in PBS with 0.1% Triton X-100 (PBS-T). Primary antibodies were diluted in blocking buffer and incubated overnight at 4°C. Samples were equilibrated to room temperature for 1 hr next morning, followed by 3× washing and 2 hr incubation with appropriate secondary antibody. For labeling proliferating cells at S-phase, BrdU (0.1 mg/g body weight) was intraperitoneally injected 2 hr prior to retina collection. For neuronal birthdating, BrdU (0.1 mg/g body weight) was injected twice in the selected postnatal date - one in the morning at approximately 10 am, the other in the afternoon at approximately 4 pm. Retinas were collected at P20. Primary antibodies and their working dilutions used in this study were as follows: mouse anti-Phospho Histone-3 (PH3; cell signaling #9706; 1∶300), rat anti-Bromodeoxyuridine (BrdU; abcam 6326;1∶400), sheep anti-Chx10 (Exalpha x1180p; 1∶300), rabbit anti-PKCα (Sigma P4334; 1∶400), rabbit anti-cleaved Caspase3 (cell signaling #9664; 1∶200), mouse anti-Pax6 (Chemicon MAB 5552; 1∶400), and rabbit anti-β-gal (cortex 03A24; 1∶7000). Nuclei were counter-labeled with TOPRO-3 or Propidium Iodide (PI).

For flat-mount immunofluorescence of PH3 labeling, eyeballs were fixed with 4% PFA for 15 min, dissected into anterior and posterior halves, post-fixed for 1 hr, washed with PBS, blocked in 2% BSA, 5% normal goat serum in PBS-T for 1 hr at room temperature. Antibody labeling was processed the same as described for cryosections. Both the anterior (for peripheral retina) and posterior (for central retina) halves of the retinas were mounted for confocal image collection.

Images were captured using a Zeiss 510 confocal microscope. The laser power setting, gain and offset parameters were optimized in the initial experiments and remained unchanged for retinas from all genotypes. For retinal radial sections, images were taken from central and peripheral two distinct regions. The central region was defined by approximately 10–15% along the distance from the optic disc or from the center point when the retina does not have an optic disc. The peripheral region was collected as guided by the edge of the retina. The reason for sampling from two distinct regions of the retina is that, during retinogenesis, the central and peripheral portions of retina represent different developmental stages [17]. Each image was stacked from six 220 µm×220 µm optical sections with 1 µm intervals to generate a 220×220×5 µm volume. Cells within this volume were identified by their positive immunostaining, and manually counted. Except for the apoptotic rate of bipolar cells, the absolute number of each cell type at different developmental stages was presented and analyzed.

### Statistical analyses

Cell counts from six independent individual retinas, collected from 6 pups of at least 3 different litters (with the exception of birthdating experiment, which used only 3 pups), were averaged to represent data of one stage of each genotype. To compare the means among 4 genotypes at each time point, one-way ANOVA was performed with post hoc Tukey multiple comparisons. The independent samples *t*-test was used for comparing data from only two genotypes. Statistical significance is defined as that p-value equal to or less than 0.05.

## Results

### Loss of rod bipolar cells in the Atoh7^−/−^ retinas occurs after they have been generated

**Figure 1:**
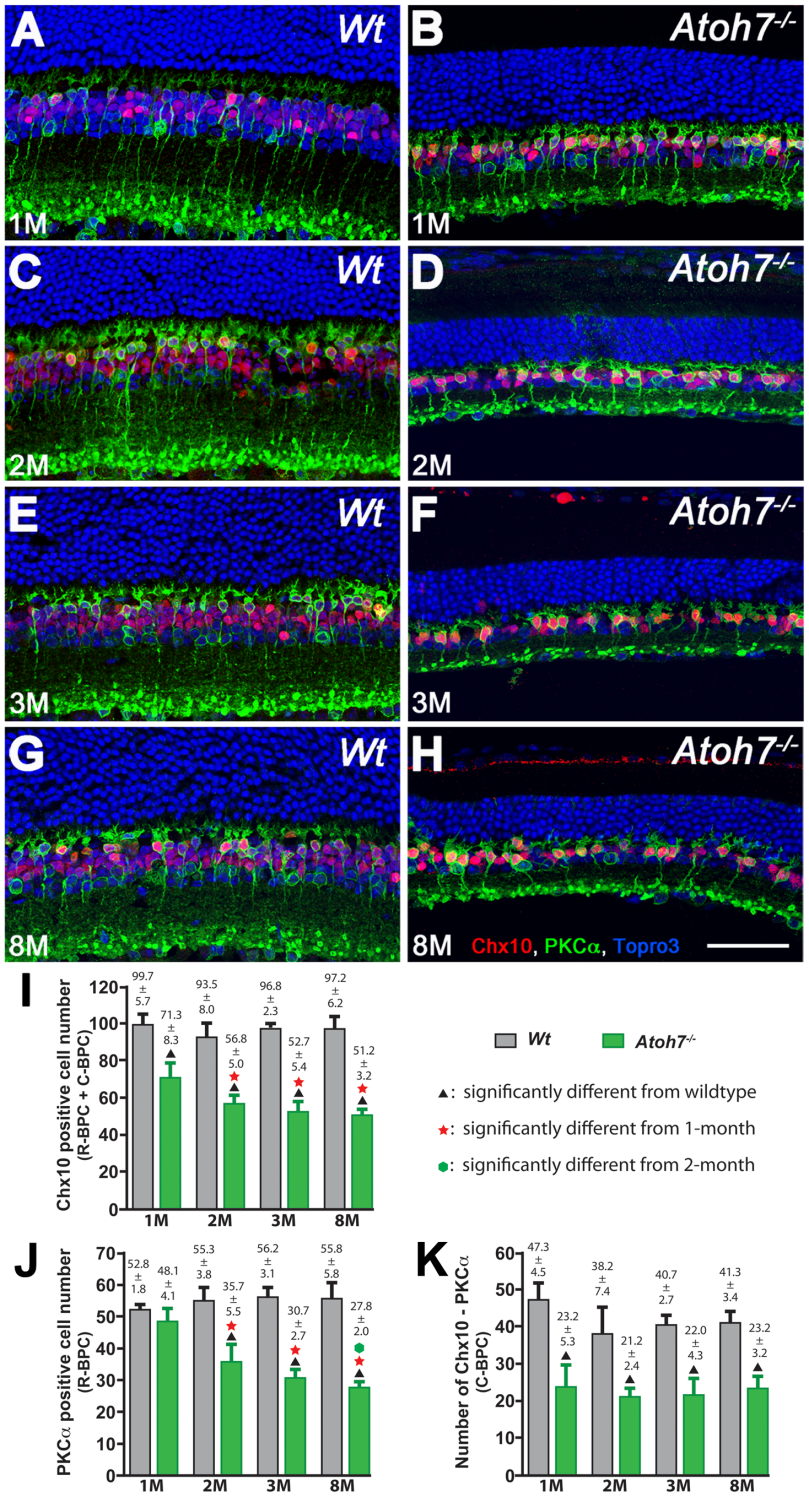
Studies in retinas at stages extended from normal development show reduction of R-BPC number occurs with aging in the *Atoh7^−/−^* retina. A–H. Confocal images of *Atoh7^−/−^* retinas displaying a normal R-BPC (red nuclei with green soma & processes) number at 1-month old (1 M); and progressive reduction of R-BPC numbers at 2 M, 3 M, and 8 M. R-BPCs maintain a constant number through all examined stages in wildtype retinas. The number of C-BPCs (red nuclei without green soma) in the *Atoh7^−/−^* retina is apparently reduced compared to the wildtype at 1-month, but the number remains unchanged with age. Red: Chx10; green: PKCα; blue: Topro3. Scale bar  = 50 µm. I–K. Histograms of R-BPC and C-BPC estimates indicating the number of R-BPCs, but not C-BPCs, are reduced with age.

Previous studies on the production of R-BPCs in Atoh7-deficient retinas have generated differing results. A couple studies showed that there was a reduced number of R-BPCs in the *Atoh7^−/−^* retina [10], [11], while others showed that the R-BPC number in the *Atoh7^−/−^* retina was normal [8], [9]. However, we noticed that different sampling ages were used in these studies. We also observed extremely thin retinas in aged *Atoh7^−/−^* mice, prompting the hypothesis that the apparent defective *Atoh7^−/−^* retina undertook a degenerative path. Therefore, the discrepancy among different studies could have been results of honest reports on the continuously degrading retinas. To test whether there was a loss of R-BPCs in the *Atoh7^−/−^* retinas after they reached maturation, we labeled retinas with PKCα and compared their numbers at the ages of 1-month, 2-month, 3-month and 8-month. Results showed that there was progressive R-BPC loss in the *Atoh7^−/−^* retina (Fig. 1). No significant difference in the number of R-BPCs was detected between the *Atoh7^−/−^* retina and the wildtype retina at 1-month of age. Starting from 2-month, the *Atoh7^−/−^* retina had significantly fewer R-BPCs than the wildtype retina at all examined ages. We did not detect any significant differences between different ages of the wildtype retinas. In contrast, the *Atoh7^−/−^* retinas exhibited a significant reduction of R-BPCs after the first month with a sharp drop between 1-month and 2-month and a slower subsequent decrease. By the age of 8-month, *Atoh7^−/−^* retinas contained only about 50% of the normal R-BPC number. To test whether neuron degeneration was an isolated phenomenon to R-BPCs or a common event to all BPCs, antibody to Chx10 was used to estimate C-BPC number in the same retinal sections. C-BPC counts were estimated by subtracting PKCα-positive R-BPC numbers from Chx10-positive total BPC numbers using a previously established method [18]. Interestingly, amid R-BPC degeneration in the *Atoh7^−/−^* retina, we did not detect any decline of C-BPCs, despite their initial number was significantly lower. Results indicated that in the *Atoh7^−/−^* retina, R-BPCs were born at a normal number, but they quickly degenerate. In contrast, the number of C-BPCs was initially significantly lower but remained stable through the adulthood. These findings not only explain the cause of a standing controversy regarding the R-BPC number in the *Atoh7^−/−^* retina but also indicate that production of C-BPCs and R-BPCs are differentially controlled.

### Vsx1 marks an attenuated C-BPC subpopulation in the Atoh7-deficient retina

**Figure 2:**
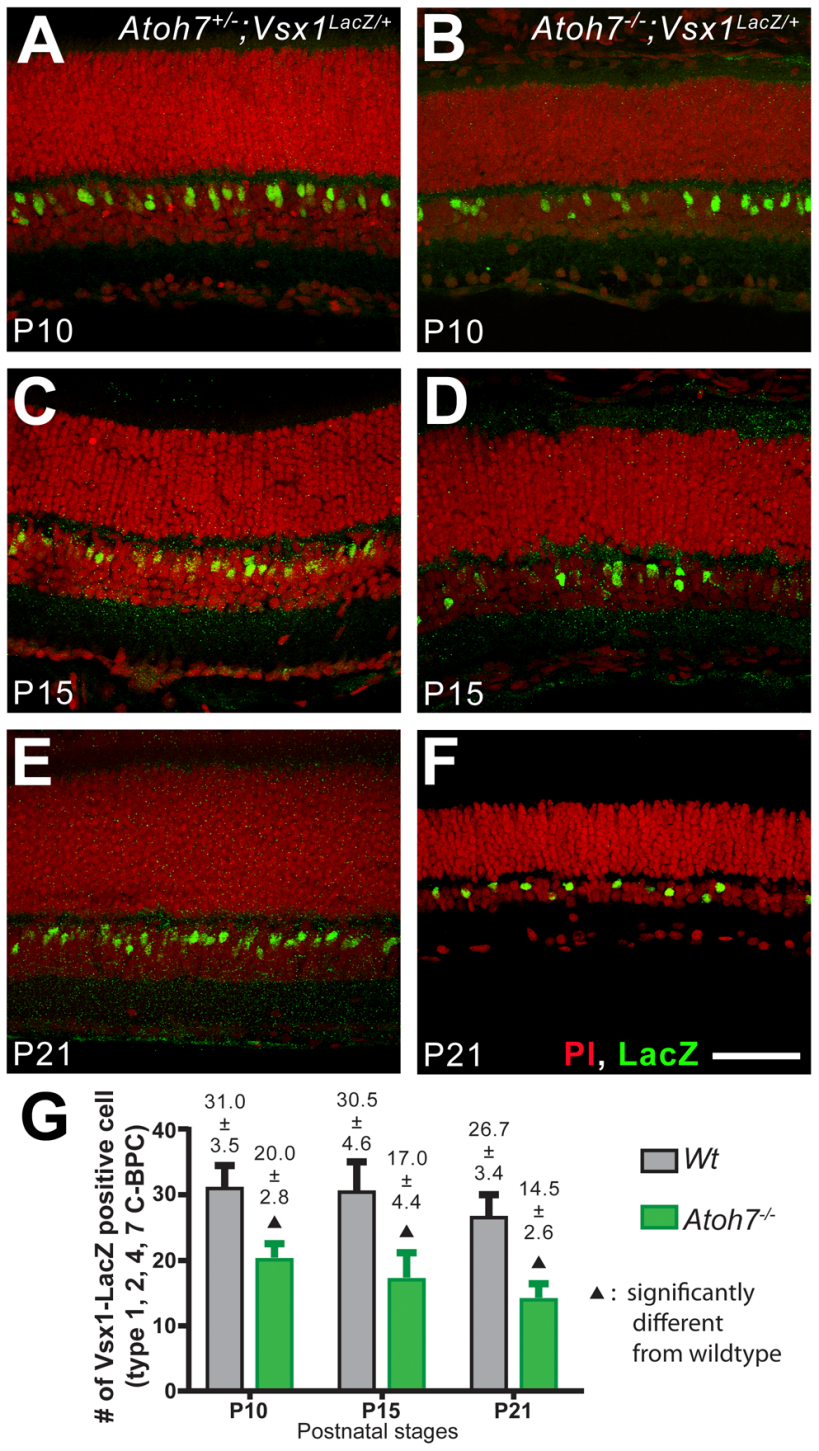
The number of *Vsx1*-expressing cells supports that fewer C-BPCs are found in the *Atoh7^−/−^* retina. A–F. Confocal images of cryosections showing *Vsx1^LacZ/+^*-expressing C-BPCs (green) in the wildtype (*Atoh7^+/−^;Vsx1^LacZ/+^*) and *Atoh7^−/−^* (*Atoh7^−/−^;Vsx1^LacZ/+^*) retinas. Green: LacZ; red: propidium iodide. Scale bar  = 50 µm. G. A histogram comparing the mean number of *Vsx1^LacZ^*-positive cells in wildtype and *Atoh7^−/−^* retinas.

To independently verify that there was a reduced number of C-BPCs in the *Atoh7^−/−^* retina, Vsx1 expressions in the *Atoh7^−/−^ and Atoh7^+/−^* retinas were compared. Among all available markers that can be used for unambiguously counting, Vsx1 is known to mark the largest subsets of C-BPCs [13], [19]–[21]. Vsx1 is specifically expressed in three OFF and one ON cone bipolar cell [21]. However, the Vsx1 antibody failed to provide countable staining patterns in our hands. Therefore, a *Vsx1^LacZ/LacZ^* mouse line was used for its robust LacZ expression in the nucleus of Vsx1-expressing cells [13]. The *Atoh7^−/−^;Vsx1^LacZ/+^* mice were created to examine *Vsx1* expression in the *Atoh7^−/−^* retinas and the *Atoh7^+/−^;Vsx1^LacZ/+^* retinas were used as wildtype controls. Retinas were collected at P10, P15, and P21 and retinal sections were labeled with antibody to LacZ. Results showed significantly reduced numbers of Vsx1-expressing cells in the *Atoh7^−/−^;Vsx1^LacZ/+^* retinas compared to the *Atoh7^+/−^;Vsx1^LacZ/+^* wildtype controls at all examined ages (Fig. 2). We noticed that, despite a lack of significant difference, the means of *Vsx1^LacZ^*–positive cells in both *Atoh7^−/−^* and wildtype retinas became smaller when the retinas were maturing. This may reflect the retinal thinning and planar spreading of cells during the normal maturation process. Results independently confirm that there is a reduced number of C-BPCs in the *Atoh7^−/−^* retina as early as P10, but this number maintains at a stable level through the maturation process.

### Birthdating shows that fewer C-BPCs but a normal number of R-BPCs are produced in the Atoh7-deficient retina

**Figure 3:**
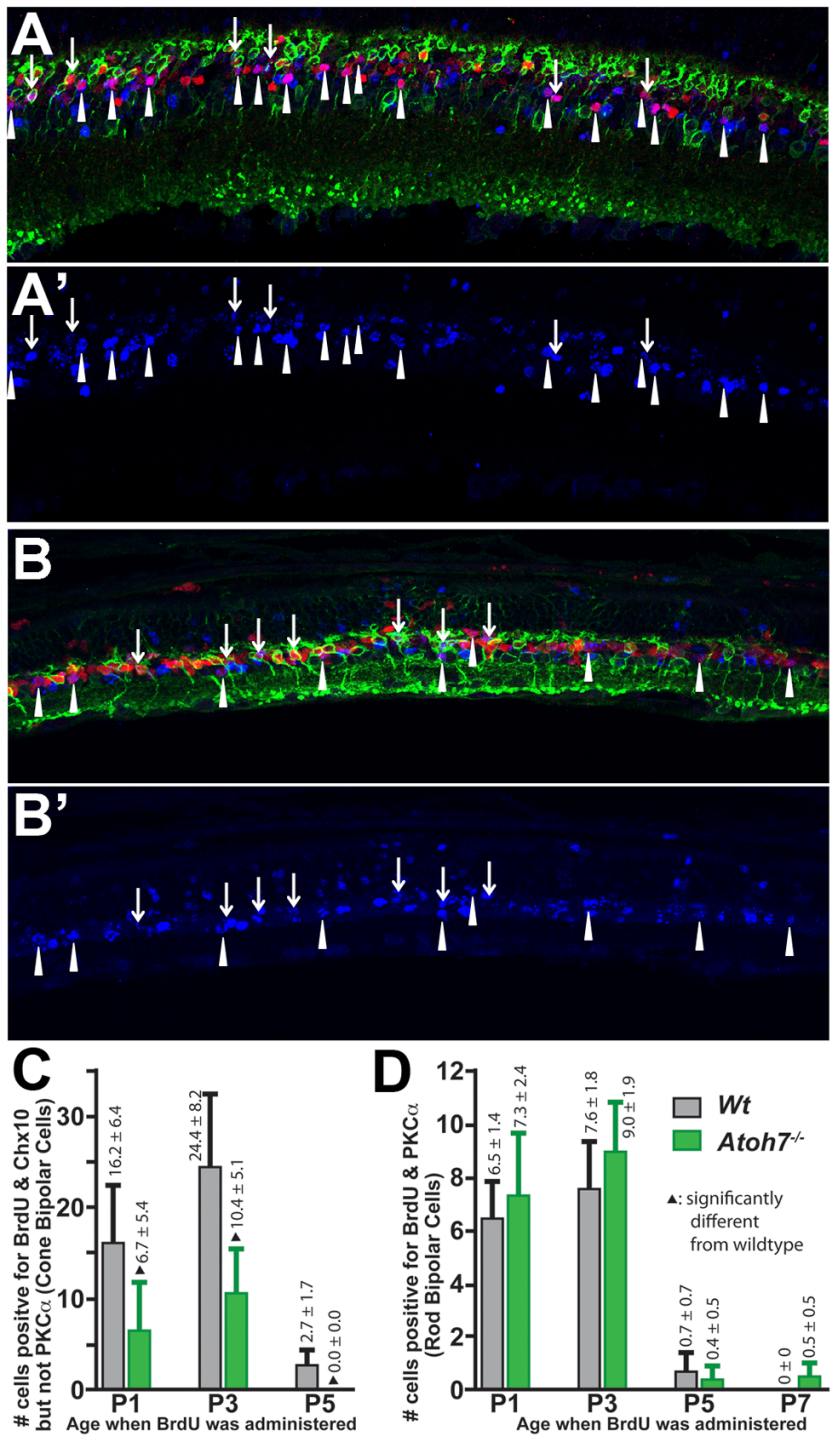
Birthdating experiments confirm fewer C-BPC and normal R-BPC production in the *Atoh7^−/−^* retina. A, B. Representative confocal images of retinal cryosections from mice that received BrdU injection at P1 and harvested at P20. In the inner nuclear layer, Chx10 antibody (red) labels both R-BPCs and C-BPCs; PKCα antibody (green) labels only R-BPCs; and BrdU (blue) marks the cells born at P1. A′ and B′ show only the BrdU channel at a positions corresponding to A and B. Arrows indicate R-BPCs and arrowheads indicate C-BPCs. Scale bar  = 50 µm. Cells outside of the inner nuclear layer were not analyzed. Images of retinas with BrdU injected at other time points are not shown. C, D. Statistical analysis comparing C-BPC (C) and R-BPC (D) cell counts that were born on a specific day. Significantly fewer C-BPCs were born in the *Atoh7^−/−^* retina on each examined day. In the sampling area, no C-BPCs were observed in either wildtype or *Atoh7^−/−^* retinas born at P7.

Bipolar cell birth peaks at around P3 and ends at around P10 [16]. A relatively low but stable C-BPC number found in the *Atoh7^−/−^* retina as early as P10 prompted the hypothesis that, different from R-BPCs, fewer C-BPCs were produced in the *Atoh7^−/−^* retina compared to the wildtype retina. To test this, a birthdating experiment was carried out. Pups at the ages of P1, P3, P5, or P7 received intraperitoneal injection of BrdU twice (∼10 am & ∼4 pm of each selected postnatal date) and retinas were collected at P20. BrdU co-labeling with Chx10 but not PKCα marked C-BPCs born at the injection day. BrdU co-labeling with Chx10 and PKCα marked the birth date of R-BPCs. Other BrdU positive cells lacking Chx10 or PKCα representing other late born cells (i.e., rods and Müller glia) were counted but not presented because they were not related to the current study. Results showed that fewer C-BPCs (<50%) were generated at P1, P3, P5 in the *Atoh7^−/−^* retina and no C-BPCs were generated in both *Atoh7^−/−^* and wildtype retinas in the examined region at P7 (n = 3). In contrast to C-BPC birth, R-BPCs in the *Atoh7^−/−^* retina were generated in relatively normal numbers if not at an increased level (Fig. 3). Results independently verify that R-BPCs are produced normally but C-BPCs are produced in a much lower number in the *Atoh7^−/−^* retina.

### Numbers of proliferating cells are reduced at the time of BPC birth and the reduction corresponds to RGC loss

Neuronal production is often referred to as cell “birth” as neurons are specified at, or immediately preceding, their exit from the last cell cycle [22], [23]. Therefore, if fewer C-BPCs were born in the *Atoh7^−/−^* retina, one would expect reduced cell proliferation being detected at the stages of C-BPC production. To further substantiate the result that fewer C-BPCs are born in the *Atoh7^−/−^* retina, we employed three different methods to compare the numbers of proliferating cells in the *Atoh7^−/−^* and wildtype retinas. In addition, the *Atoh7^−/−^* retina has 95% of RGC loss and RGCs are required for retinal histogenesis [7], [9], [24]. We believe reduced C-BPC production in the *Atoh7^−/−^* retina is a secondary non-cell autonomous effect of RGC loss but not a result of missing the Atoh7 protein cell autonomous function because *Atoh7* is normally not expressed in, nor required for, the bipolar cells and their progenitors [25]. To test this, we added two mouse lines, *Pou4f2^−/−^* and *Atoh7^−/−^;Pou4f2^−/−^*, in the comparison. The *Pou4f2^−/−^* retina has fewer RGC loss (∼80%) and the *Atoh7^−/−^;Pou4f2^−/−^* retina has more RGC loss (∼99%) compared to the *Atoh7^−/−^* retina. We reason that proliferating cell numbers should reflect the existing RGC numbers if loss of RGCs, but not loss of genes, is the cause of reduced C-BPC production. For simplicity the *Atoh7^−/−^;Pou4f2^−/−^* retina will be regarded as DKO herein.

To cover the majority of the BPC production time window, retinas were collected at P0, P4, P7, and P10. Three center most cryosections, as judged by their largest circumference and presence of optic disc (the DKO retina does not have a visible optic disc, therefore was only judged by the largest circumference), were labeled with Phospho Histone-3 (PH3) and Bromodeoxyuridine (BrdU) antibodies to detect cells at M-phase and S-phase respectively. For BrdU labeling, BrdU was administered 2 hr prior to retina collection. The same antibodies were also used to label the flat-mounted retina for independent estimations. However, BrdU on flat-mounted retinas was later aborted because an objective counting was not achievable in our hands. To aid analyses, image data from the central retina and the peripheral retina were focused separately.

**Figure 4:**
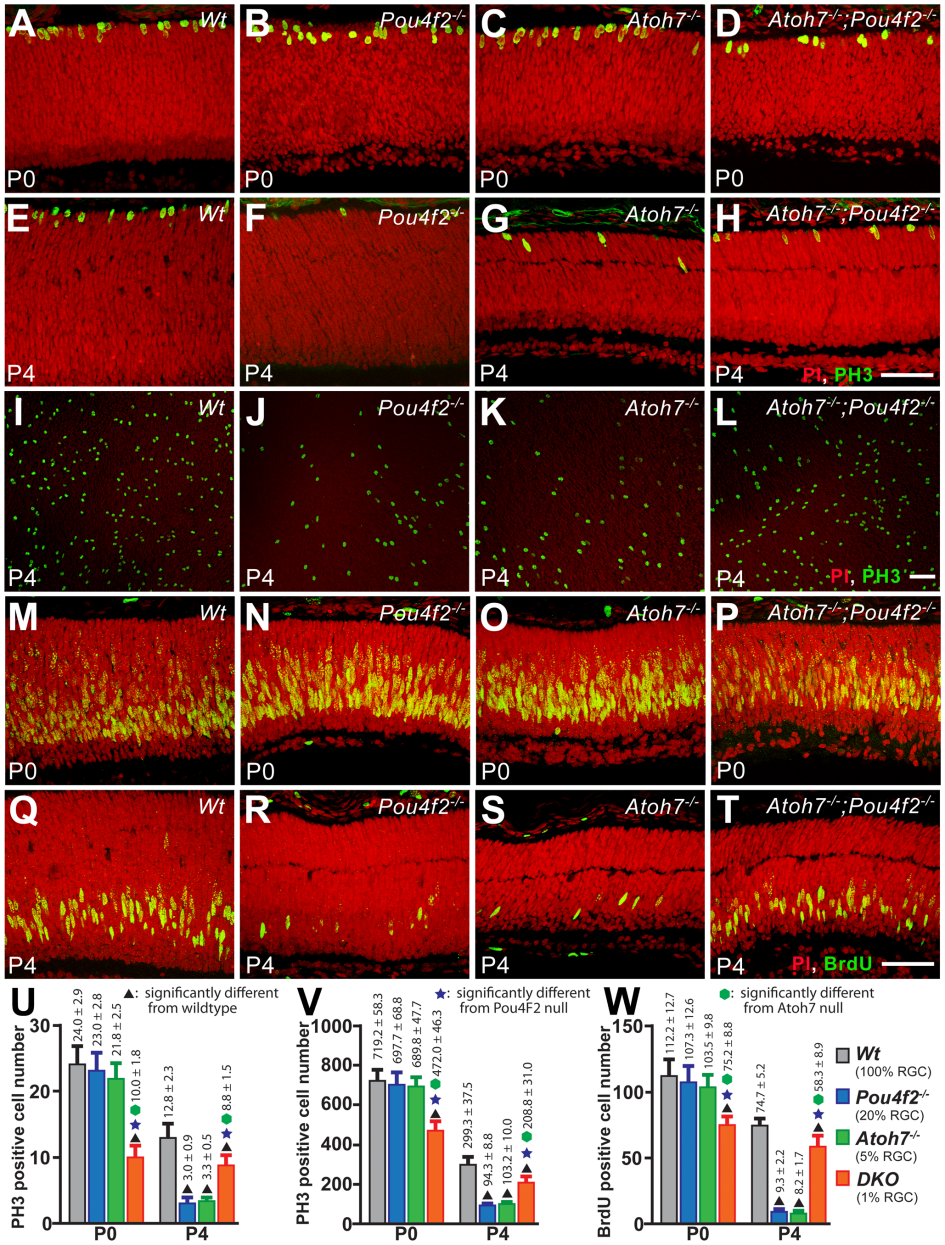
Phospho-Histone-3 and BrdU labeling show unexpected proliferation dynamics in the central retinas that have varying degrees of RGC loss. A–H. Representative confocal images at P0 and P4 show PH3-positive cells (green) in cryosections of wildtype and RGC-depleted retinas. I–L. Representative confocal images at P4 show PH3-positive cells in flat-mounted retinas. M–T. Representative confocal images at P0 and P4 show BrdU-positive cells (green) in cryosections. Red: PI. All scale bars  = 50 µm. U–W. Histograms comparing the numbers of proliferating cells estimated by PH3-positive cells in sections (U), PH3-positive cells in flat-mounts (V), and BrdU-positive cells in sections (W). No proliferating cells were found in the sampled region in all examined retinas at P7 and P10.

In the central retina, the number of proliferating cells in neither *Pou4f2^−/−^* nor *Atoh7^−/−^* was significantly different to the wildtype at P0 but were both significantly lower than the wildtype at P4, the peak BPC production time. The DKO retina exhibited interesting proliferation dynamics in the sampling region: it was drastically lower than all other genotypes at P0, but became significantly higher than *Pou4f2^−/−^* and *Atoh7^−/−^* while remaining lower than the wildtype at P4 (Fig. 4). It appeared that the number of proliferating cells in the DKO retina decreased at a much slower rate compared to all other retinas. No proliferating cells were found in the central region in all retinas by P7. We added the PH3 positive cell numbers of P0 and P4 to roughly represent the cumulative proliferating cell number. Results showed that wildtype >*Pou4f2^−/−^*≈*Atoh7^−/−^*>DKO in both sectioned and flat-mounted retinas. However, adding P0 and P4 BrdU positive cells showed Wildtype >*Pou4f2^−/−^*≈*Atoh7^−/−^*≈DKO. We have no concrete explanation for the reason why the BrdU method provided results that differed from the PH3 method. However, we believe the proliferating cell number detected by BrdU reflect PH3-positive cells at a later timepoint. BrdU labels cells at S-phase, especially at early and mid S-phase. Cells at late S-phase are often not detectable due to insignificant BrdU incorporation. The time required to complete the cell cycle, thus from S-phase to M-phase, becomes longer during late retinogenesis. Therefore, cells in S phase that would be labeled by BrdU at P0 and P4 would likely be detected as PH3-positive cells at later stages, e.g. P1 and P5. We observed, from the PH3 labeling results, that proliferating cells in the DKO retina did not decline as quick as in the *Pou4f2^−/−^* and the *Atoh7^−/−^* retinas. Therefore, the relatively high accumulated number (P0+P4) of BrdU-positive cells in the DKO retina may actually reflect the abnormally higher number of proliferating cells at a later stage. To avoid further confusion elicited by the unique cell proliferation dynamics in the central DKO retina, here we take only the PH3 data into consideration, which presents the accumulated proliferating cell number in the central retina as wildtype >*Pou4f2^−/−^*≈*Atoh7^−/−^*>DKO.

**Figure 5:**
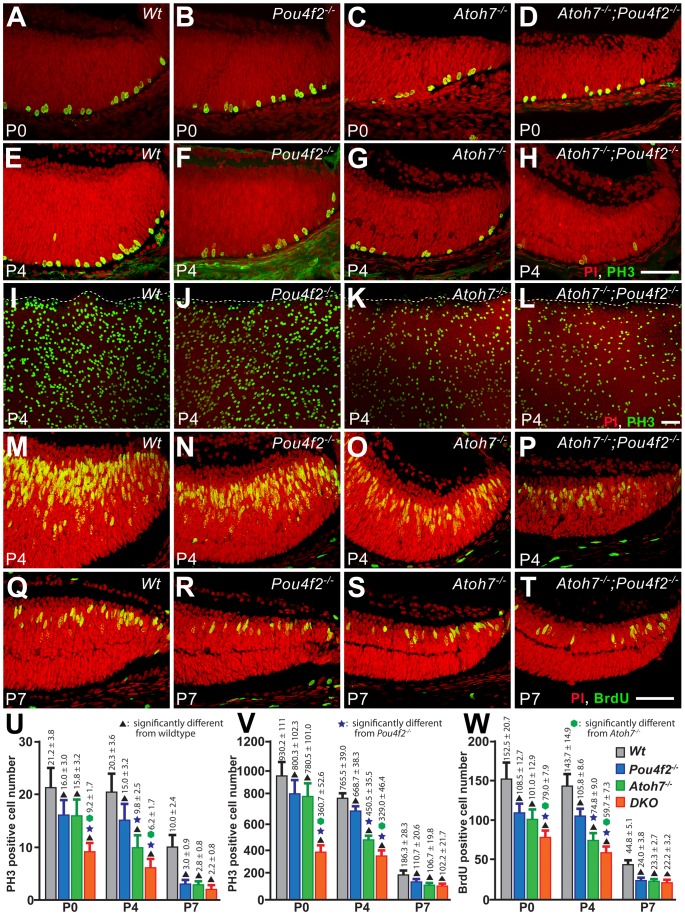
Phospho-Histone-3 and BrdU labeling of the peripheral retinas show the number of proliferating cells corresponds to the existing RGC number. A–H. Representative confocal images at P0 and P4 show PH3-positive cells (green) in cryosections of wildtype and RGC-depleted retinas. I–L. Representative confocal images at P4 show PH3-positive cells in flat-mounted retinas. Dash lines mark the edge of the retina. M–T. Representative confocal images at P4 and P7 show BrdU-positive cells (green) in cryosections. Red in all panels: propidium iodide. All scale bars  = 50 µm. U–W. Histograms comparing the numbers of proliferating cells estimated by PH3-positive cells in sections (U), PH3-positive cells in flat-mounts (V), and BrdU-positive cells in sections (W). Proliferating cell numbers found in all retinas at P10 are either zero or close to zero. There is no significant difference between any two genotypes at P10.

Birth of BPCs begin prior to P0 despite peaking at ∼P3, P4. A retinal neurogenesis wave ripples from the center towards the periphery and development of peripheral retina is about 3 days behind the center of the retina [17]. Therefore, to better represent BPC birth, we used the same PH3 and BrdU labeling methods and looked into cell proliferation in the peripheral retina. Results showed that the numbers of proliferating cells in the peripheral retina correspond to the numbers of existing RGCs (Fig. 5). The most striking timepoint was at P4, around the peak BPC production time, when the number of proliferating cells in each peripheral retina was significantly different from all others, showing wildtype >*Pou4f2^−/−^*>*Atoh7^−/−^*>DKO. The accumulated proliferation of the whole retina was estimated by combining data from central and peripheral retinas of all examined stages: this result corresponds to the amount of existing RGCs in the retina showing wildtype >*Pou4f2^−/−^*>*Atoh7^−/−^*>DKO. Collectively, by examining retinas with varying degrees of RGC depletion, our results show that the amount of proliferating cells corresponds to the existing RGC number. Therefore, the observed low C-BPC production in the *Atoh7^−/−^*, and other RGC-depleted retinas is most likely a non-cell autonomous result of RGC-depletion but not a direct cell autonomous result of gene loss.

### The wildtype retina has more apoptotic BPCs than the RGC-depleted retinas

**Figure 6:**
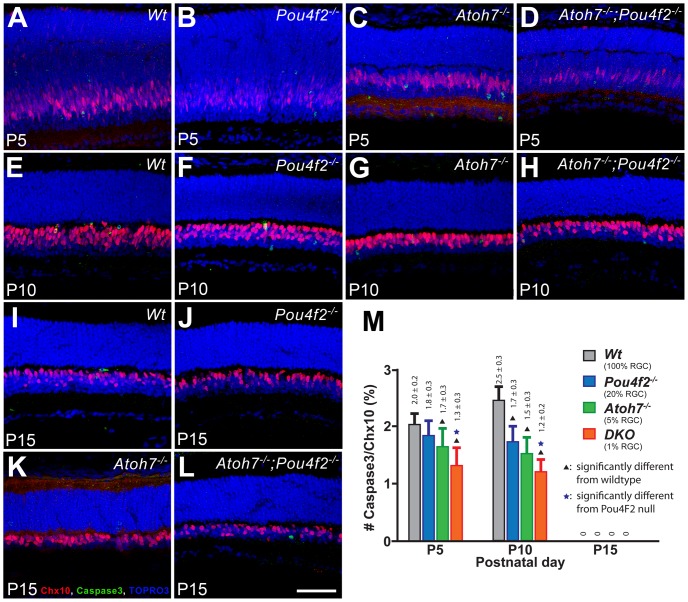
Assessment of apoptotic BPCs show a higher rate of apoptotic BPCs in the wildtype retina. A–L. Representative confocal images of the central retinas from wildtype, *Pou4f2^−/−^, Atoh7^−/−^*, and DKO mice at P5, P10 and P15. Retinas were labeled with antibodies to Chx10 (red), cleaved Caspase-3 (green) and TOPRO-3 (blue). Scale bar  = 50 µm. M. A histogram showing the percentage of Caspase-3 and Chx10-double positive cells over the Chx10-positive cells, depicting the ratio of apoptotic bipolar cells over all bipolar cells in four different retinas.

There are two possibilities for the observed low C-BPC numbers in the RGC-depleted retinas. First, C-BPCs were indeed insufficiently produced. Secondly, C-BPCs quickly die after a normal number of these cells were generated. If the latter is true, more apoptotic BPCs should present in the RGC-depleted retinas. To test this, we labeled the bipolar cells and apoptotic cells with antibodies to Chx10 and cleaved Caspase-3 at P5, P10, and P15. P5 was selected for being a stage when bipolar cell production had just passed the peak and the sampling area had only a few Chx10-positive progenitor cells that could complicate the analysis. P10 was selected for being the stage when bipolar cell production reached an end. P15 was selected for the stage when retinal histogenesis becomes complete [26], [27]. Caspase-3 positive cells overlapping with Chx10 signals were counted and normalized with the total number of Chx10- positive cells. In contrast to our prediction that there should be no difference in the number of apoptotic cells, results showed that the wildtype controls had actually more apoptotic bipolar cells than any of the RGC-depleted retinas (Fig. 6). At P5, the *Atoh7^−/−^* (5% RGC) and the DKO (1% RGC) retinas had a significantly lower BPC apoptotic rate compared to the wildtype. At P10, all RGC-depleted retinas had a significantly lower rate of BPC apoptosis compared to the wildtype. At P15, no Chx10-positive apoptotic cells were found in any retinal samples examined. The mean of the apoptotic rate showed a correlation between the existing RGC number and the BPC apoptotic rate. The retinas with the lowest RGC number displayed the lowest level of apoptotic BPCs (Fig. 6). This result indicated that the reduced number of C-BPCs in the RGC-depleted retinas during late retinal histogenesis was not a result of the death of specified bipolar cells. This confirms that the reduced C-BPC numbers in the RGC-depleted retinas reflects a reduction in the birth of C-BPCs.

### Fewer C-BPCs, but a normal number of R-BPCs, are produced in retinas with reduced RGC numbers

**Figure 7:**
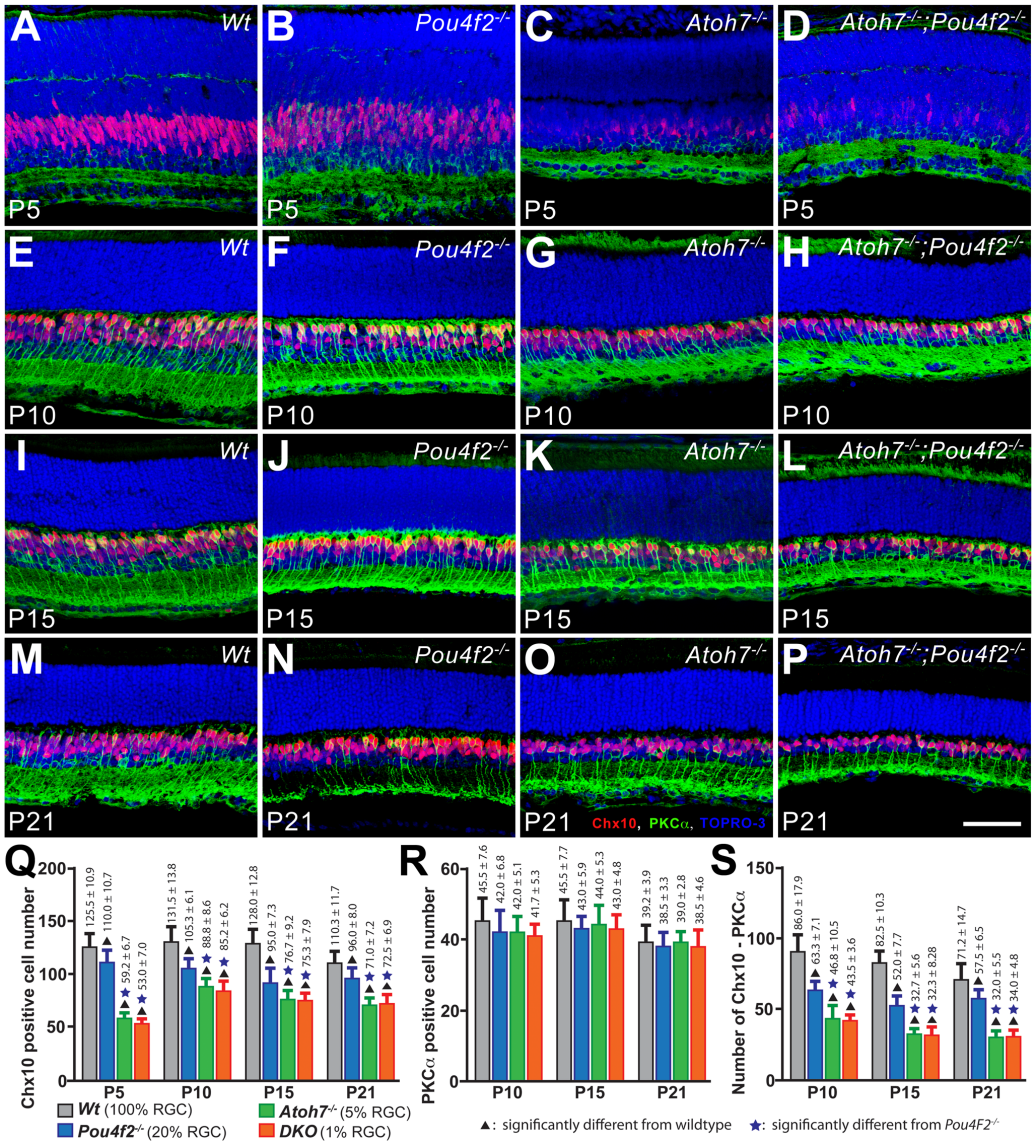
Assessment of C-BPC and R-BPC numbers show reduced C-BPC numbers and normal R-BPC numbers in the RGC-depleted retinas. A–P. Representative confocal images from the central retinas from wildtype, *Pou4f2^−/−^, Atoh7^−/−^*, and DKO mice at P5, P10, P15, and P21. Samples were labeled with Chx10- (red) and PKCα- (green) to depict total BPCs and R-BPCs respectively. Nuclei were counterstained with TOPRO-3 (blue). Scale bar  = 50 µm. Q–S. Histograms comparing the mean numbers of total BPCs (Q), R-BPCs (R), and C-BPCs (S) in four different retinas with varying degrees of RGC depletion. Means of the C-BPC populations were calculated by taking the difference between Chx10- and PKCα- positive cell counts.

Results of the apoptosis study demonstrate that there are fewer dying BPCs in the RGC-depleted retinas. The results of PH3 and BrdU labeling experiments show that retinas containing fewer RGCs produce fewer cells during late retinogenesis when BPCs are born. Together, these results indicate that fewer BPCs are born in the retinas with fewer RGCs. However, the birth of BPCs overlaps with rods and Müller glia and the birth of R-BPCs is apparently not affected in the *Atoh7^−/−^* retina. To address how C-BPC and R-BPC production were affected in an RGC-depleted retina, we again labeled total BPCs with antibodies to Chx10 and R-BPCs with PKCα at P5, P10, P15, and P21 in cryosections from retinas with varying degrees of RGC depletion. *Chx10* is expressed in all bipolar cells as well as retinal progenitor cells. To avoid confusion between bipolar cells and progenitor cells, we collected data only from the central retina starting at P5 when the numbers of progenitor cells in the area are minimal. As presented earlier, C-BPC counts were estimated by subtracting PKCα-positive R-BPC numbers from Chx10-positive total BPC numbers. Results showed a strong correlation between the numbers of Chx10-positive cells and the amount of existing RGCs (Fig. 7). At P5, when BPC production has just passed its peak, all three RGC-depleted retinas had significantly fewer Chx10-positive cells than the wildtype (Fig. 7A–D & 7Q). There was no significant difference between the *Atoh7^−/−^* retina (5% RGC) and the DKO retina (1% RGC), but they both had significantly fewer Chx10-positive cells than the *Pou4f2^−/−^* retina (20% RGC). In the sampling area, there are only a handful of progenitor cells, which is negligible when compared to the number of bipolar cells at this stage. Therefore, differences in Chx10-positive cell numbers should represent differences in total bipolar cell numbers. Progenitors cease to exist in the sampling region prior to P7 (Fig. 4), and thus there are no possible Chx10-positive progenitor cell related artifacts after this stage. At P10, P15, and P21 the trend of the change in Chx10-positive cell numbers were identical to P5, showing wildtype >*Pou4f2^−/−^*>*Atoh7^−/−^*≈DKO (Fig. 7Q). It is noticeable that there is no significant increase between P5 and P10 in both *Pou4f2^−/−^* and wildtype retinas, indicating BPC production in the sampling region has mostly completed by the time. However, the Chx10-positive cell number significantly increased between P5 and P10 in the *Atoh7^−/−^* and DKO retinas (compare P5 and P10 in Fig. 7Q), indicating a delayed BPC production in these two retinas. We believe this delayed BPC production is due to Atoh7-deficiency because of the common genotype between *Atoh7^−/−^* and the DKO retinas while the *Pou4f2^−/−^* retina does not have delayed BPC production. It is possible that these delayed BPCs are mostly R-BPCs because the total BPC number at P5 is only marginally higher than the C-BPC number at P10 (compare P5 in Fig. 7Q with P10 in 7S) suggesting C-BPC production may have also reached an end in the *Atoh7^−/−^* and DKO retinas. However, further experiments are required to provide a conclusive answer.

PKCα was labeled for estimating R-BPC numbers in the same set of retinal sections. At P5, PKCα just started to appear in a few R-BPCs (Fig. 5A–D). The diffuse labeling pattern prevented us from obtaining a precise counting at this stage. The staining pattern became clear at P10 (Fig. 7E–H). Surprisingly, no significant difference in the R-BPC number were observed among all retinas at all examined stages (Fig. 7R). It appears that, despite there is a possible time shift of R-BPC production, the number of R-BPCs is not affected by RGC reduction. Therefore, the differences in Chx10-positive cell numbers may solely be attributed to the differences of C-BPC numbers. Together, there were approximately 70% of C-BPCs produced in the *Pou4f2^−/−^* retina and 50% of C-BPCs produced in the *Atoh7^−/−^* and the DKO retinas compared to the wildtype. Our results show that, in RGC-depleted retinas, C-BPC production is significantly reduced but R-BPCs are born in a normal number.

## Discussion

In this study, we re-examine the *Atoh7^−/−^* retina and show that rod bipolar cells (R-BPCs) are produced at a normal number but cone bipolar cells (C-BPCs) are produced at a lower number. The normally produced R-BPCs quickly degenerate between 1-month and 2-month of age, after which degeneration slowly proceeds. In contrast, the insufficiently produced C-BPCs are maintained at a stable number through out adulthood. By including two other RGC-depleted retinas, we further show that differential regulation of R-BPC and C-BPC production is a result of RGC loss and is unlikely a result of loss of Atoh7's cell autonomous function. We conclude that the number of C-BPC being born is related to the number of RGCs in the retina, but the total number of R-BPC being born is independent of RGCs.

### Gene loss or cell loss

During retinogenesis, genetic networks are tightly intertwined. Knocking out a transcription factor often elicits a cascade of events and a series of phenotypes. Some phenotypes are related to the primary cell autonomous function of the missing factor that often leads to missing or aberrant cell types. Other secondary phenotypes may appear following the initially missing or over-produced cells. Therefore, using knockout mice to study gross cellular events in a given tissue presents a potential dilemma in deciphering the results. It is difficult to distinguish whether the observed phenotype is the primary effect of loss of the protein's cell autonomous function or the secondary effect elicited by the altered cell population. However, by comparing multiple knockout mice that have a similar alteration of cell populations, the primary cell autonomous and the secondary non-cell autonomous effects can be dissected. Here, we provide three arguments that suggest, rather than a direct effect of losing Atoh7 and/or Pou4f2 protein function, our observations of reduced cell proliferation and reduced C-BPC production are secondary effects caused by RGC loss. First, neither of these genes is directly involved in promoting cell cycles: *Pou4f2* is expressed after the final cell cycle and thus cannot be involved in any aspect of cell cycle regulation. Therefore, the reduction in cell proliferation observed in the *Pou4f2^−/−^* retinas is more reasonably attributable to a secondary effect of the loss of RGCs. Atoh7 appears at S-phase of the last cell cycle before a cell is committed to become a neuron [28]–[31]. It is believed to be involved in assisting newborn cells to exit the cell cycle [25]. Therefore, the direct effect of Atoh7 deficiency can only cause an increase of proliferating cells [25] and cannot cause a reduction in proliferating cells. Hence, the observed reduction of cell proliferation in the Atoh7-deficient retina should be a secondary effect. Second, the selected knockout gene products are not involved in BPC production. It is well known that, in the retina, *Pou4f2* is only expressed in RGCs and their precursors, but not in BPCs or their immediate precursors [15], [32]–[34]. Therefore, Pou4f2 cannot be directly required for BPC's fate determination. The expression of *Atoh7* is downregulated at the time of BPC birth and it is not found in the cell lineage that give rise to BPCs [8], [10], [25]. There is no apparent or logical role for Atoh7 in BPC production. Therefore, the observed truncated C-BPC productions in the *Pou4f2^−/−^*, *Atoh7^−/−^*, and DKO retinas are not directly related to gene loss. Third, reduction of cell proliferation and C-BPC production corresponds to the extent of RGC loss further strengthening our argument that reduced C-BPC production is the result of RGC shortage but not specific gene deletion.

### Production of C-BPCs vs. other retinal neurons

In contrast to a reduced C-BPC production that is associated with the existing RGC number, we believe, as previously addressed by many colleagues, a population increase in other cell types in the *Atoh7^−/−^* and *Pou4f2^−/−^* retinas are cell autonomous consequences of the missing proteins. It has been shown in the *Atoh7^−/−^* retina that amacrine cells, cone cells, and horizontal cells are increased [8], [10], [35]. It is believed that *Atoh7^−/−^*-expressing cells transdifferentiate into other early retinal cell types at the expense of RGCs [8], [10], [35]. It has also been shown that *Pou4f2^−/−^*-expressing cells become amacrine-like cells at the expense of RGCs [34]. The relatively thicker inner nuclear layers found in the *Pou4f2^−/−^* and DKO retinas at P5 observed in this study (compare Fig. 7B to 7A and 7D to 7C) are in agreement with previous reported amacrine cell overproduction found in the Pou4f2-deficient retina [34]. These aberrantly produced amacrine-like cells quickly die prior to P10.

Our results show that production of R-BPCs is not affected by either loss of genes (*Atoh7* and/or *Pou4f2*) or loss of RGCs. R-BPCs do not form direct synapses with RGCs in the retinal circuitry. Instead, R-BPCs synapse with AII (A2) amacrine cells [36]. It is, therefore, tempting to speculate that formation of R-BPCs may be related to amacrine cells. However, R-BPCs are produced normally in a amacrine cell depleted retina that contains an excessive number of RGCs [37]. Therefore, R-BPC generation is independent from either amacrine cells or RGCs. Despite R-BPC's normal generation in RGC-depleted retinas, their survival requires an intact retina. Currently, we do not have enough information to speculate whether R-BPC degeneration is directly linked to insufficient RGCs or to the loss of other cell types (e.g. amacrine cells) due to insufficient RGCs. However, we think R-BPC degeneration is accelerated in retinas that have suffered a further RGC reduction. This view is supported by our previous results that show the R-BPC number in the DKO retina (1% RGC) is already significantly lower than the wildtype at 6-week of age, when such reduction is not yet detectable in an *Atoh7^−/−^* retina [9].

### Unanswered questions and obstacles

The results of this study provide the initial suggestion that production of C-BPC and R-BPC are differently regulated. However, our results do not predict possible molecular candidates that are involved in C-BPC and R-BPC fate decision. Emerging evidence shows that retinal progenitors are heterogeneous [38]. Therefore, it is possible that C-BPCs and R-BPCs are from distinct progenitor pools and hence responding to RGC shortage in different manners. It is also possible that RGC shortage affects the fate choice of BPC precursors resulting in a normal number of R-BPCs being produced at the expense of C-BPCs. An early marker to separate newborn C-BPC and R-BPC is needed to address these unanswered questions.
